# The experiences of medical students with ADHD: A phenomenological study

**DOI:** 10.1371/journal.pone.0290513

**Published:** 2023-08-22

**Authors:** Megan Godfrey-Harris, Sebastian Charles Keith Shaw

**Affiliations:** 1 Brighton and Sussex Medical School, Brighton, East Sussex, United Kingdom; 2 Department of Medical Education, Brighton and Sussex Medical School, Brighton, East Sussex, United Kingdom; Singapore General Hospital, SINGAPORE

## Abstract

Attention Deficit/Hyperactivity ‘Disorder’ (ADHD) is a form of neurodivergence, characterised by lifelong differences in attention, impulsivity, and hyperactivity. University students with ADHD underachieve academically and tend to have lower levels of self-esteem. Medical schools have an obligation to minimise barriers for students with ADHD. Understanding the experiences of medical students with ADHD is vital to promote inclusive approaches. Our exploratory research question was: “What are the experiences of medical students with ADHD?” This was an interpretive phenomenological study. Loosely structured interviews were conducted with participants (medical students with ADHD) over Zoom. Subsequent transcripts were analysed using interpretive phenomenological analysis. Six people participated. Our analysis identified the following themes: Identity and diagnosis; ADHD profile; system issues; conflict, competition and compensation; improving the experience. Participants reported experiences of bullying and isolation at medical school, perpetrated by doctors and peers, as well as feelings of alienation when unable to conform on placement and in exams. From this, participants adopted survival strategies, such as masking, to avoid being ostracised. All recognised their ADHD status when their mental health deteriorated during their medical studies. Of those who disclosed their diagnosis, none were offered personalised support. Participants feared disclosure, largely due to weaponised professionalism and the effects of toxic competitiveness in medicine. They yearned for a sense of belonging. Participants reported strengths associated with ADHD such as empathy and working well under pressure, which are highly desirable aptitudes for doctors. This study has highlighted areas where medical schools can be instrumental in cultivating an environment where medical students with ADHD can thrive, not just survive. This may take the form of peer support groups, alongside reasonable adjustments throughout medical school–particularly for Objective Structured Clinical Examinations, for example. Enabling these students to thrive may help to prevent early burnout and subsequent attrition from medicine.

## Introduction

Under the neurodiversity paradigm, Attention Deficit/Hyperactivity ‘Disorder’ (ADHD) is a form of neurodivergence, alongside others such as autism or dyslexia [1]. More specifically, ADHD is a neurodevelopmental label, characterised by lifelong differences in attention, impulsivity, and hyperactivity [2]. Differences may become challenges when mismatched with external factors, such as the sensory environment or differing communication styles. As such, challenges experienced can be pervasive across social, educational and/or occupational settings [3]. The prevalence of ADHD in UK adults is estimated at 3–4% [3], with a male-to-female ratio of 3:2 [4]. Prevalence in UK medical students has been found to be at least 5.5% [5]. ADHD diagnostic rates vary, influenced by factors such as gender, race, socioeconomic status, and ethnicity [6]. Students who achieve high grades in academia, such as those needed for entry to medical school, are at a particularly high risk of missed diagnosis of ADHD [7].

Current diagnostic criteria for ADHD are reliant on identifying deficits in function, consistent with the medical model of disability [8]. This enables identification of possible challenges and implementation of subsequent supports, such as medication and psychological therapy. However, deficit-based diagnosis ignores strengths such as increased energy, cognitive dynamism, and hyperfocus [9, 10]. Cognitive dynamism describes ceaseless mental activity, which facilitates adaptability, innovation, and heightened problem-solving abilities [9]. Consideration of challenges without consideration of such associated strengths acts to reduce the experience of ADHD to a single dimension, overlooking both intersectionality and more holistic profiles. Furthermore, framing of differences as deficits is dependent on context and socio-environmental expectations, as reflected in the neurodiversity paradigm [11].

Considering external expectations is particularly important within a medical context. The wider literature is replete with studies arguing that the culture of medical schools is both toxic and competitive [12]–particularly for neurodivergent students [13]. Thus, those appearing socially different to the majority may risk falling foul of this culture through their observed differences. In the case of neurodivergent medics, this has taken the form of bullying and isolation from their peers, leading to a sense of learned helplessness [14, 15]. Furthermore, the learning process itself is important to consider here. Whilst explicit education remains paramount, medical education continues to rely on social and experiential learning through the hidden curriculum [16]. This hidden curriculum underlies medical studies in a pervasive fashion, and through this, medical students are expected to absorb the wider culture of medicine–helping to transform them into the next generation of doctors [16]. However, due to their communication differences, it is possible that medical students with ADHD may be at a further disadvantage here. Previous research has certainly supported this assertion in the case of autistic medical students [14].

More broadly, university students with ADHD have consistently been shown to underachieve academically [17] and to have lower levels of self-esteem [18]. Therefore, students with ADHD may require psychological and social support services, in addition to academic coaching and adjustments, to achieve their full potential [19]. For example, to ensure the different domains of ADHD are supported, interventions such as cognitive behavioural therapy (CBT) are emerging as useful tools, having been shown to improve executive functioning and wellbeing [20]. Improved mental wellbeing has, in turn, been shown to have positive effects on intrinsic motivation, engagement with university, and academic success [19]. However, it is difficult to know how medical students with ADHD can be holistically supported to thrive in medical school without the input of the students themselves.

Giving voice to the experiences of students from marginalised groups is a fundamental step towards achieving social justice. These groups are often discredited and dismissed when sharing their experiences–a concept known as epistemic injustice [21]. In the case of neurodivergence, access to such stories may also prove helpful for neurodivergent students/trainees through positive role modelling, promoting the celebration of difference and acceptance of oneself [22]. Research over recent years has led to the inclusion of dyslexic, dyspraxic, and autistic voices within the literature [23–25]. However, what of the voices of those with ADHD? Searches identified no studies exploring the experiences of people with ADHD in medicine, nursing, or any other health professions. Learning may be impacted by differences in working memory and executive functioning similarly to other forms of neurodivergence [26]. However, differences in social domains, motivation and arousal/regulatory systems unique to ADHD are also likely to have an impact [27, 28]. Understanding the experiences of medical students with ADHD is therefore vital to promote inclusive approaches to education, assessment, and practice. For example, under the UK Equality Act (2010), medical schools have an obligation to make adjustments to reduce barriers for medical students with ADHD [29]. However, whilst the UK General Medical Council (GMC) has provided guidance with broad examples of potential adjustments for disabled students [30], a lack of research into the experiences of medical students with ADHD means little is known regarding which, if any, of these may be useful. To that end, our research question was: “what are the experiences of medical students with ADHD?”

## Materials and methods

### Methodology

This qualitative study used an interpretive phenomenological approach–seeking to explore and interpret lived experience through the perspectives of the participants [31]. An interpretive phenomenological approach endeavours to find deeper meaning and understanding of experiences in relation to the subjective world around us [32]. This is achieved by acknowledging and embracing the researchers’ insider perspectives to interpret the meanings within participants’ narratives, leading to a dynamic and intersubjective elucidation of the findings [32]. Gadamer described this concept as a “fusion of horizons” [33]. Heidegger, who developed interpretive phenomenology, suggested that humans cannot exist separately from the world around them and, therefore, to understand the everyday and authentic experiences of humans, we must consider the wider cultural, social, and political contexts surrounding us [34]. Thus, assuming an ontological approach to phenomenology [35].

### Conception and positioning

The first author of this paper, Meg (MGH), is a fourth-year medical student. She was diagnosed with ADHD at the end of her second year of medical school.

*“Hi*, *I’m Meg*! *I’m a 4th year medical student*. *Forgetful*, *fidgety and fickle*. *These were the embarrassing aspects of my personality that I found most difficult to deal with until I was diagnosed with ADHD between second and third year of medical school*. *I spent more time worrying about seeming “unprofessional” than I did engaging with teaching”* (MGH–first author).

Meg’s interest in this study stemmed from her own experiences of being signposted to support for dyslexic students in the absence of conclusive support for those with ADHD. This study itself was born of her own experiences, following discussion with Seb (SCKS), the second author of this paper, who is a doctor and researcher with ADHD. Seb is also autistic and dyslexic.

*"Hi*, *I’m Seb*! *I’m a lecturer in medical education*. *I came late to my ADHD diagnosis*, *during my specialty training*. *The lack of ADHD voices in the literature led me to not recognise this important aspect of my own intersectional identity*. *Diagnostic criteria aside*, *I attribute many of my strengths to my ADHD*. *For example*, *my creativity and resourcefulness have proved to be a blessing within the world of qualitative research”* (SCKS–second author).

Prior to the study, Meg reflected on her own experience, facilitated by Seb. This reflective process enabled her to acknowledge and contextualise her preconceptions and feelings so that they could be used to positively influence the project [36]. This is particularly important in interpretative phenomenology when these perspectives are used to guide the study and the analysis of results [36].

### Ethical approval

This study was approved by the BSMS Research Governance and Ethics Committee (reference: ER/BSMS9E80/2).

### Recruitment

A purposive sampling approach was used. Following gatekeeper approvals, a recruitment advertisement was emailed to all medical students at a single medical school in the South of England. People who were interested were invited to contact Meg by email. They were then sent a participation information sheet and consent form to read. Informed consent was received and audio-recorded at the start of each interview.

### Data collection

Drawing on the wider literature, Meg created an interview topic guide (see S1 Appendix) iteratively with Seb, using their insider experiences. Meg then conducted loosely structured interviews with participants over Zoom. These lasted about an hour each. Broad topic areas included:

- Learning environments
- Emotional and social experiences
- Disclosure
- Learning about ADHD at medical school
- Medication

Interviews were audio-recorded, transcribed, and encrypted using Zoom’s internal software. Once Meg had reviewed and corrected the transcripts to ensure they were verbatim, they were stored securely on the university OneDrive.

### Data analysis

The transcripts were analysed using the approach of Smith, Flowers and Larkin to the interpretive phenomenological analysis framework [37]. This involved Meg fully immersing herself in the data by closely reading the transcripts multiple times and making notes alongside them. Smith, Flowers and Larkin emphasize the importance of highlighting distinctive phrases and emotional responses [37], which Meg did physically on paper using pens. Next, Meg examined the notes to search for prevalent and poignant themes for each individual participant, called Personal Experiential Themes (PETs). These PETs were then compared, seeking conceptual similarities that allowed the themes to be constructed into Group Experiential Themes (GETs) across all participants. This reflects the hermeneutic circle in which the transcripts are interpreted in relation to the context, and the context is analysed in relation to the transcripts [38]. Revising the themes based on their congruence with the transcripts, and in discussion with Seb, enabled an authentic narrative of the experiences of the participants, and the impact of these experiences [37]. This was an iterative process over several months, with ever-refined changes following re-discussion and reanalysis to ensure each constructed PET and GET reflected participants’ experiences.

## Results

Six individuals participated (Table 1). Participants have been given pseudonyms to personify their narratives.

**Table 1:** Demographic information and interview durations.

Pseudonym	Duration of interview in minutes	Age	Gender identity	Year of medical study (out of 5)	Time of recognition	Resits / retakes
Zara	35	23	Cis-female	5	4^th^ year	Verbal case presentation, Objective Structured Clinical Examination (OSCE)
Emma	90	25	Cis-female	5	3^rd^ year	None
Amar	74	22	Cis-male	4	2^nd^ year	Verbal case presentation
Taylor	58	20	Non-binary	3	1^st^ year	None
Kate	63	24	Cis-female	5	4^th^ year	Verbal case presentation
Tiah	48	24	Cis-female	4 (forced to take a year out)	Intermission in 4^th^ year	OSCE, forced to intermit halfway through year 4

Five GETs were constructed (Table 2).

**Table 2:** Group Experiential Themes and subthemes.

Group Experiential Theme	Subthemes
Identity and diagnosis	Identity, social media, recognition of ADHD, benefits of diagnosis
ADHD profile	Positives, communication, mental health, emotional understanding
System issues	Teaching, OSCEs, adjustments and helplessness, hidden costs
Conflict, competition, and compensation	Bullying, hostility on clinical placements, surviving, professionalism, peer interactions, competitiveness, shamefully perfect
Improving the experience	Engagement and accountability, reducing minority tax, community, role modelling

### GET1: Identity and diagnosis

Participants identified with their ADHD uniquely, and whilst some embraced it wholeheartedly, others found aspects challenging. Acquiring a formal diagnosis was tortuous, but participants valued the advantages it brought, including validation.

#### 1a: Identity

Participants felt ADHD was an intrinsic and inseparable part of their identity. Zara explained “*you’ve always had it*. *So*, *you don’t know what you would be like if you didn’t have it*.*”* This was a double-edged sword, as some feared their ADHD identity could be quickly removed at a formal psychiatric assessment if found to not have ADHD. Kate explained “*I was so scared because I was like oh my god*, *what happens if I don’t have it*… *I was petrified*.*”* They recognised the contextual nature of ADHD, dependant on the environment and the person. Zara reflected *“it’s not one size fits all… everyone’s experience is going to be different*.*”* Some felt there should be a shift towards identity-first language. Emma explained *“I don’t think I have ADHD*. *I think I am an ADHD person*.*”* For Amar, the chronicity and perceived lack of positives of ADHD was difficult.

Kate explained the impact of ADHD on her identity in medicine: *“people love like the good characteristics of ADHD… But I feel like there’s a lot of stigma with… the bad side of ADHD… there’s still so many negative connotations of ADHD in med school*. *And I think there’s a lot of gaslighting of it*, *especially for women”*. Some were subsequently apprehensive about their future in medicine. Emma explained “*a lot of the medical training isn’t suited to people with different… ways of thinking*, *with neurodivergence let’s say*.*”*

#### 1b: Social media

Social media had a notable effect on participants’ identities. Increased awareness brought positives and negatives. The depathologisation of ADHD was harmful. For example, Amar explained *“ADHD is downplayed quite a lot… it’s like how people say… ‘everyone’s a little bit ADHD’”*. Identifying with portrayals of ADHD on social media was challenging, particularly the superpower rhetoric. Emma explained *“apparently*, *it’s big on social media*, *so I think it’s less stigmatized*, *but it doesn’t feel that way… I feel like they very much glamorize or romanticize the things that I struggle with… I clearly have something else*. *I’m clearly more broken somehow*, *more incapable somehow*.*”*

However, in the absence of an open culture at medical school, participants found affirmation in hearing similar experiences on social media. It was helpful for suggesting potential coping strategies–both for study and life management–as well as signposting support and treatment outlets. For example, Taylor explained how it has been helpful for them: “*hearing people’s experiences online made me think “Oh*, *that sounds like me*. *I should get assessed” … [I’m now implementing] lots of like study hacks that I saw on TikTok or Youtube”*.

#### 1c: Recognition of ADHD

Participants had difficulty recognising and identifying with their ADHD due to stereotypes of laziness, unintelligence, and disorganisation. They also noted how the stereotype of ADHD only affecting young boys was a hindrance. Emma explained “*as a woman with ADHD*, *nobody had*, *I guess*, *caught on that this could be what it was because I was high achieving*.*”* Differing presentations of ADHD in gender could have been a barrier to diagnosis. Kate argued *“I think that’s just because we’re way better at hiding our symptoms*, *and we’re kind of able to slightly use them more to our advantage*.*”* Participants experienced diagnostic overshadowing, whereby receiving diagnoses of anxiety and depression meant they were not further investigated, despite unresolved challenges at medical school.

Some had not realised how vastly their experiences differed to their neurotypical peers until their ADHD was recognised. Kate explained “*it was honestly like ground-breaking*, *because I genuinely thought everyone was like me”*. The impact of their ADHD status not being recognised earlier was felt profoundly. Kate explained *“it’s like… a mourning for kind of everything you struggled with when you were younger*.*”*

#### 1d: Benefits of diagnosis

The main benefit of diagnosis was the professional validation of their experiences and struggles. Zara explained, “*you get like confirmation that it’s just that’s how your brain works*, *you’re not just being lazy or being stupid*, *which is quite validating*.” Formal diagnosis was helpful in validating their need for medication. Kate described “*diabetes is a deficit of insulin… ADHD is a deficit of dopamine*. *And I found that so useful in… accepting the condition… I physically am lacking in something*.*”* The diagnosis was also instrumental for participants’ self-discovery journey. Kate explained “*the diagnosis made me understand myself better and like made me kind to myself… I feel more in control of who I am*.” As well as being kinder to themselves, participants felt better at empathising with others, including patients. Amar explained *“I’ve definitely appreciated ADHD a lot more… so I think that can definitely be a positive um*, *especially… when I was doing psychiatry*.*”*

### GET2: ADHD profile

Participants reflected on their strengths and challenges. They felt there were several advantages of having ADHD, as well as areas of medicine being well-suited to individuals with ADHD. However, challenges were experienced throughout medical school, affecting their relationships, mental and physical health.

#### 2a: The positives–multidimensional

Participants felt their ADHD led them to be more inquisitive, lateral thinkers. “*Thinking outside the box and questioning*”, as Taylor put it. Being highly empathetic was noted to be a positive, enabling them to easily build rapport with patients. Emma explained “*the way that I respond and react to emotional things… I think it helps me better relate to patients… And I think I’m quite bubbly… which makes people comfortable”*.

Participants all described their ability to hyperfocus for extended periods of time. Additionally, they work most efficiently under pressure. This was beneficial for studying and working in the National Health Service (NHS). Amar explained, “*I am able to focus quite a lot… get work done*, *especially when I’m enjoying it… when I get started*, *and when I’m in the zone I can keep going for hours”*. Participants felt that their dynamic learning methods would benefit neurotypical people, and feedback from peer teaching had shown this. Kate explained “*the ways we learn to like compensate it are so fool proof that*, *like it just works for everyone”*.

Some reflected on how ADHD can be advantageous for medical work, especially in emergency medicine. Taylor explained *“I knew it would always like be a stimulating environment*. *And like*, *I love learning*. *And I love talking to people”*. Kate explained *“I love it*, *and I think my ADHD really suits me [in the emergency department]*. *I’m really empathetic and intuitive… I’m able to adapt easily to different scenarios”*.

#### 2b: The challenges–communication

Participants felt a mismatch in communication between themselves and surrounding neurotypical people. Participants’ body language was often misinterpreted, causing tension. Emma explained “*It doesn’t look like I’m listening because I need to do multiple things at once to keep engaged… a lot of people have gotten very angry*”. Participants felt their effort to match the communication of their neurotypical peers was not reciprocated. Kate explained *“it’s really difficult actually to turn around to someone… to say to them actually that really hurt me*, *or actually that’s really insensitive… people can be very stubborn about it”*.

#### 2c: The challenges–mental health

No participants were diagnosed with ADHD prior to reaching breaking point in medical school. For most, this came in their clinical years, when they experienced the greatest number of transitions–an absence of continuity and increased responsibility for orchestrating their learning without accountability. Kate explained *“I think*, *because of so much content*, *so many new people*, *just so many new environments*. *It just overwhelmed me… There was no kind of safety in anything*”. Emma felt that medical school had been the catalyst for her diagnosis, explaining “*if I wasn’t in medical school I would never have been in that situation where everything felt out of control and unsupported”*.

In a cyclical nature, participants felt their mental health deteriorated in the absence of support, but that help was inaccessible with the normalisation of poor mental wellbeing at medical school. For example, Emma repeatedly reached out for help, but experienced discounting: “*they were like ‘this doesn’t sound like you’re struggling with anything more than a normal medical student would be struggling with’*”. Participants spiralled without support, unable to study with their deteriorating mental health. However, some precluded themselves from accessing vital ADHD support from the medical school and mental health services due to confidentiality concerns. Amar said *“how anonymous is something that’s offered by the medical school*. *Uh that I would be a bit sceptical of”*.

#### 2d: The challenges–emotional understanding

Participants reflected on how their strong emotive responses have been challenging, especially when not shared by neurotypical peers. Kate explained, *"I’ve had to learn… sometimes my friends don’t mean to be mean*. *They don’t realize what they’re doing is bad”*. Whilst hyper-empathy was viewed as an advantage, it was also problematic for some. Emma was aiming for a less patient-facing specialty because “*I struggle with like separating my emotions*. *I think that I react very strongly to the way that other people are feeling*. *Um*, *and I take it home with me*, *and I ruminate on it*”.

### GET 3: System issues

Participants examined how they were disabled by the environment around them, rather than an intrinsic impairment. Zara explained *“people with neurodivergent conditions aren’t lesser*, *it’s just society*.*”* Throughout medical school, participants felt they had to overcome additional hurdles to survive.

#### 3a: Teaching

Experiences of lectures felt ableist. This educational approach was physically restrictive and unengaging, placing participants at disadvantage. Kate explained *“I used to hate lectures*, *just sitting there—horrible… I literally had no idea half the time what they were saying*.*”* But with mandatory attendance, participants had no choice but to attend and compensate later for missed content. Zara explained *“if any lectures aren’t recorded*, *that’s a nightmare*, *because you even if you’re trying… if you’re paying attention as much as you can throughout the lecture you’ll never like*, *it just doesn’t pick up as well*.*”* Kate reflected, *“we know that this stupid*, *old-fashioned way of teaching just is not productive and examining people*, *and yet we still continue to do it*.*”*

Participants felt that the medical school expected an unreasonable amount of flexibility for last minute timetable changes. When this was combined with participants’ innate challenges with organisation, they suffered overwhelming stress. Emma explained *“having to be so flexible*, *I find really difficult*, *and it makes me really stressed… there’s no empathy or sense of understanding as to how much this can actually impact somebody like me’s life”*. This was exacerbated difficult when participants had additional appointments, essential for maintaining their wellbeing.

#### 3b: OSCEs

OSCEs were particularly disabling for participants, largely due to inflexibility in timings and the structure of these assessments. Kate describes her most recent OSCE:

*“I felt completely alienated. I felt different, and I knew I had a disability in that moment… all the instructions were verbal. All the questions were verbal. I couldn’t mouth read anyone. I couldn’t, you know, just sit there, and stop for a minute, everything was on the clock… I’ve had to ask them to write out the question, because I don’t know what they’re asking. And they’ll write that down the feedback that would be like ‘oh, you were struggling to like, understand the question’… Because it’s too much noise, and it’s like that auditory processing… The fact you can’t ask certain questions. You can’t check things. No OSCE I’ve been in has been alike to any clinical scenario I’ve ever been in.”*

Participants felt their communication style was a disadvantage in exams, with most having failed at least one spoken assessment (Table 1). The OSCEs were a key example of this. Kate examples that examiners “*notice I’m like a bit fidgety… but they think it’s like anxiety*. *I’m like no*, *it’s just me… it’s because I’ve got ADHD*, *and I’m fidgety*.” Echoing such issues, Zara reflected:

*“You just appear less kind of calm about your responses and… the way you speak is less, um, structured. So, it seems like you’re less confident… definitely fidgeting and structuring answers is always something that comes up, and it’s something I really struggle with, because to me my answer does seem structured. But to them they’re like ‘Oh, no, you’re just jumping about everywhere’”*.

#### 3c: Adjustments and helplessness

Participants reported a lack of true supports, in favour of keeping up appearances. Emma illustrated “*I think the medical school like to sort of claim that they’re inclusive and supportive and do reasonable adjustments… but I don’t always think that it’s true*.*”* Taylor reflected “*the school’s offered nothing for ADHD in terms of support*.” Participants were offered extra time and extensions to deadlines, regardless of helpfulness. Kate explained “*I wish it was more personalized in the learning plan*. *We’re all different*, *and we all have different needs*.” Participants’ repeated efforts in getting more support were met with resistance by their medical school. This led to helpless states, whereby participants stopped trying to advocate for adjustments. Emma explained “*it’ll be like pulling teeth to get that information from the med school*” when asking for exam guidance. Participants attributed these negative responses to cost or feelings that their concerns were seen as insignificant.

Participants felt a responsibility to spend time and resources advocating for their needs–if they did not, then who would? Some tried but were met with discounting and disbelief. Kate explained, “*I know I’m very privileged*, *being like cisgender*, *like heterosexual woman*, *white… I feel really comfortable going in and being like*, *‘okay*, *I struggle with this thing*, *and I want to talk about it’… Every day you’re having to say*, *if not twice a day*, *you’re having to go up to someone and explain why you are the way you are… And then you also have that extra like emotional burden of having to take on the fact that probably 80% of people aren’t going to listen to you*, *or fully take on what you’re saying”*.

Stonewalling led participants to feel helpless. Amar explained “*It’s just up to their judgment of what it is they decide to attribute to ADHD and what they don’t*, *uh*, *rather than necessarily based on your personal experience*.*”* Kate explained “*we’ve all been having to cope with it so long*. *We know what helps us”*.

#### 3d: Hidden costs

Costs associated with being in a minority student group, known colloquially as ‘minority tax’, refers to uncompensated efforts and responsibility to reduce inequalities between themselves and their peers. There was a minority tax associated with having ADHD at medical school, taking its toll in time, money, energy, and emotions. Travel was particularly costly, especially in placements far from home. These involved distance from their usual support systems and unfamiliar and/or complex journeys. Emma explained, “*sometimes I think that I need to get a taxi*. *I’ll get a taxi*, *and then they won’t reimburse it because there is a bus… on the bus where it’s crowded*, *lots of different people*. *Everybody’s loud*. *Everybody is talking*, *lots of different smells*, *lots of different things happening*.*”* Zara was embarrassed when she explained *“I’ve really struggled with… traveling to kind of new places like when it’s a one off or not very frequent… Like… the planning of like the travel and things like that*.*”*

Medication alleviated many struggles, reducing unhealthy coping mechanisms such as smoking, drinking alcohol and binge eating. These improvements allowed energy and time to be diverted towards studying and engaging with placement. Whilst this was a dramatic improvement, medication cost money and was not the ‘magic bullet’ in providing equity with peers. Kate explained *“I’m not quite as focused as the normal person still*. *But it’s enough for me to cope with”*.

Taylor regularly attended therapy, enabling them to cope better with the emotional and social impacts of ADHD at medical school. However, Taylor acknowledged their privilege in being able to access multiple avenues of support privately. They felt that the medical school could play a role in achieving equity. They said, “*I think if other people have support whether that be coping strategies or therapy*, *or assessments or support groups then that would help people”*.

### GET 4: Conflict, competition and compensation

Interactions with peers and staff significantly impacted participants’ experiences of medical school. Some experienced direct discrimination against their ADHD, causing overtly negative attributions. However, experiences of additional microaggressions invariably contributed to feelings of mistrust, isolation, and internalised ableism.

#### 4a: Bullying

The experience of being bullied by doctors, particularly senior doctors, was normalised amongst participants. Emma recited her first-time history taking, *“it made me cry because I was trying really hard*, *and [the doctor] essentially told me that I shouldn’t be a doctor… [I] was reprimanded in a really cruel way”*. Taylor also experienced bullying on the ward by a consultant, they said *“I went and cried for a bit in the toilets*, *did my deep breathing*, *and then went back… we get on with our day”*. When Taylor experienced bullying, they blamed themselves for being upset, explaining *“someone who was less sensitive or maybe more resilient might have been able to handle it”*. Throwaway comments from consultants about ADHD and participants’ ability to achieve was damaging to their self-esteem. Emma explained “*that’s still made me doubt to a certain extent whether I can do this*, *and that’s something I think I’m gonna struggle with daily”*.

#### 4b: Hostility on clinical placements

New clinical placements triggered anxiety. Participants feared how new teams would receive them, embedded in feelings of personal inferiority. Zara explained “*being new on the ward or being new in a place*, *and you just don’t know how people are going to react to you… But then*, *after that*, *you’ve met them once at least*, *so you can kind of do better”*. More often than not, however, participants were simply ignored. Zara explained “*they just ignore you… it feels like there’s no point in you being there because you’re not taking anything in*.*”* When they tried to actively engage, they faced hostility. Emma explained, *“I have to be really ballsy*, *and like*, *ask a lot of questions to keep myself engaged in learning… and then the consultant will be like*, *“Why are you interrupting my ward round*? *Why are you talking*?*” But they won’t answer my questions if I ask*.”

#### 4c: Surviving

Participants developed survival strategies to avoid humiliation–rigorously self-regulating and forming a protective shield. Subsequently, survival became easier with time. Emma explained *“I’ve got like tougher skin… consultants call me stupid*, *I move on… I know a lot of the consultants… so I know how I need to react to them*.*”*

A vast amount of energy was expended masking (hiding) their ADHD. Take Taylor, for example, who said that “*I do things so people don’t comment”*, but later explained *“everything is so much easier [with medication] …and not like being so depleted of… all of my spoons”*. There was little reserve for learning when participants prioritised masking. Emma explained, “*that extra worry of… making sure that you’re not seeming distracted*, *and you’re seeming engaged*, *and you’re making the patient feel safe and comfortable and able to talk to you… that’s a lot of things to have to do all at once”*.

Exhaustion from masking on placement exacerbated executive functioning differences and thus negatively impacted motivation for self-study. As a result, basic activities of daily living, such as eating, felt insurmountable with their little remaining energy and executive functioning capabilities. Emma explained *“there are literally like a week’s worth of dishes just lying around… Like it’s a mess in here*, *and the reason it’s a mess is because I’ve got a lot going on at university*.*”*

Seemingly small mistakes significantly worsened participants’ experience of medical school, especially at regional placements. Zara explained “*I’ve forgotten my laptop*. *I’ve forgotten chargers*, *I’ve forgotten clothes*. *I like really struggle with packing week to week*, *and so often found kind of I don’t have the things that I need*.” Most experienced time blindness, and despite implementing coping strategies, they consistently arrived late. Or worse–lost marks in assessments due to not managing time ‘appropriately’. For example, Tiah explained “*I’m like pretty much late to everything*. *I always*, *always feel stressed about it… I find the whole concept of OSCEs very tricky because I struggle so much with time and organizing myself”*.

#### 4d: Professionalism

Fear of weaponised professionalism was a barrier to participants disclosing their ADHD to the medical school. Weaponised professionalism in this context refers to characteristics inherent to ADHD being perceived inappropriately as unprofessional, despite these characteristics likely bearing no true impact on their performance as medical students, or on patient care. Seeing ADHD be used to ‘prove’ incompetence of a peer deterred Amar from disclosing his diagnosis, explaining “*I have an inherent distrust of medical schools*.” There was a scepticism of the premise for collecting disability data from medical schools and the GMC. Emma explained, *“I worry that because people don’t understand it… they will not… inform me of opportunities that they don’t believe that I can do”*. Professionalism concerns had been raised relating to ADHD traits. Kate experienced a strong sense of moral injury when a consultant criticised her professionalism for consoling a distressed patient and fidgeting in clinic. Kate left, explaining “*it felt like such an attack on who I am and everything I stand for”*. She also felt her gender had a part to play in being perceived as unprofessional, explaining *“like female characteristics they just were so against”*.

#### 4e: Peer interactions

Participants were wary of disclosing their ADHD to peers, but not to people outside of medicine. As Kate explained, *“I think my ADHD characteristics [at work] has always been viewed so positively… but I find that in med school*, *I think I’m negatively treated because of it”*. Many experiences with peers centred around either overt judgement or a fear of judgement. This negatively impacted disclosure and wellbeing. Emma recalled, “*the way [peers] talk about [ADHD] before they find out that I’m like that makes me self-conscious and hyper-aware and hyper-vigilant and makes me struggle to be sociable*.*”* Kate echoed this, *“everyone just talks negative about [ADHD] which kind of pulls you down a bit”*.

#### 4f: Competitiveness

Participants attributed negative interactions with peers to a toxically competitive environment at medical school. Kate argued *“I think up till now it’s kind of been viewed*, *if you say things that are disabilities or health issues that you’re making an excuse*. *I think it’s very toxic”*. They felt there was a perceived weakness of ADHD in medicine. Amar explained “*at the end of the day… I don’t think I would get much empathy from anyone… I don’t think they would take me like*, *you know*, *um*, *seriously”*.

Some felt ashamed that they needed ADHD medication to survive medical school. Competitiveness led to likening use of ADHD medication to cheating. Emma explained, *“I think it feels a bit like cheating because you know that like the medication you take*, *other students take to study”*.

#### 4g: Shamefully perfect

A combination of feelings of inferiority and the competitive environment led to perfectionist mindsets. In turn, this further fostered masking behaviours in an attempt to hide their secret shame. Kate explained “*I’ve previously always been known to be… hyper-organised*. *[In] hindsight*, *that was me compensating”*. Participants set impossible expectations for their studies causing overwhelm and frustration. Zara explained “*when you have ADHD you don’t know what’s normal and what’s not normal*. *You just think you’re kind of like lower in the general ability compared to your peers”*.

Participants had difficulty recognising their hard work and the contributions this has to their success. They attributed success to luck, but failures to their own wrongdoings. Some felt fraudulent in having an ADHD diagnosis itself–worried they were just making excuses for not coping. Emma, for example, avoided disclosing initially due to “*suffering with all that imposter syndrome stuff and feeling like a fraud”*.

### GET 5: Improving the experience

Participants reflected on their positive experiences and how aspects of these could be applied elsewhere in medical school. They also explored how negative experiences could have been improved.

#### 5a: Engagement and accountability

Environments where participants were encouraged to engage, without fearing repercussions, were most conducive for learning. Examples included small-group sessions and simulation. Amar said, *“those are the ones I take the most from… where you get the chance to speak*.” Enjoyment on placement correlated with positive engagement from the team. Participants valued having a friendly face on the ward, such as a clinical fellow. It enabled them to quickly become comfortable in potentially hostile environments, maximising learning opportunities that may have otherwise been missed.

Participants needed accountability to stay motivated and engaged with the course. For example, some found regular contact and soft deadlines valuable. Zara explained “*just having more checking ins… I… think it’s quite useful… because it has that outside accountability”*. Their attempts at undirected studying with peers had limited success because they often digressed from the task at hand. Instead, they felt academic tutors that provided accountability would be more beneficial.

In their pre-clinical years, a checklist of lectures required for exams was beneficial for participants who had difficultly planning self-study. In the clinical years, however, participants had to fend for themselves, leaving them unable to prioritise and rationalise which knowledge was needed. Emma explained, “*they don’t provide very clear guidelines*, *or like lists of things that need to be covered*, *and so that… it gives me too much freedom to do what I actually enjoy*, *and then I end up*, *not covering the stuff I need to be covering*”. Participants found initiating self-study in the clinical years became almost impossible with the lack of routine.

#### 5b: Reducing minority tax

When participants were proactive in accessing support, this was financially and emotionally expensive. To reduce minority tax on placement, Kate suggested the use of learning support cards. She explained “*Like in the clinical years*, *I just want something that says I’ve got these things*, *this is what helps me*. Just to show that I’m not bull*******g”. It was felt that neurotypical allies could strive to make clinical spaces more inclusive for their colleagues.

#### 5c: Community

A culture of silence was isolating for participants, who then felt liberated when meeting other students with ADHD. Emma explained “*Nobody talks about it… Why don’t we talk about it*? *[Meeting other medics with ADHD] made me feel like I wasn’t so strange”*. They yearned for belonging. Taylor explained “*humans like to form communities with people who are similar to them in environments that are different and challenging”*. Participants wanted to exchange experiences with other medical students with ADHD, signposting helpful resources and ultimately providing validation. Kate explained “*I love when I get to chat to other medics with ADHD… it kind of fulfils everything I feel about myself”*. Participants with an overbearing fear of disclosure were particularly isolated. As Amar described, “*you would be the first medical student I know… I’m not aware of medical students or doctors with ADHD*.*”*

#### 5d: Role modelling

Role modelling was important for participants’ self-esteem, providing validation and encouragement. Connecting with doctors with ADHD and senior medical students provided reassurance that their dreams were achievable with ADHD, not despite it. Emma explained “*to see that there are doctors who can achieve the things that people sometimes say “Oh you maybe don’t go for that… don’t aim so high’… It was very encouraging to see that you can aim high and you can achieve it*, *and it doesn’t stop you from doing anything*”.

Some thought it would be helpful to hear how challenges of ADHD in medicine can be overcome through mentoring. Kate explained *“I’d love to go chat someone just be like ‘how is it*?*’*, *and I hope they would get paid for it as well”*, acknowledging the often-unremunerated nature of mentoring. Meeting a doctor with ADHD on placement meant Emma saw a different style of working, providing her with the confidence and permission to develop her own style of doctoring. Kate also noted the benefit of having visible ADHD role models from an early age. On her paediatrics rotation, parents informed her of the positive impact she had for being proud and unapologetically ADHD.

## Discussion

This was the first study to explore the experiences of medical students with ADHD. Whilst positives, such as cognitive dynamism and increased empathy, were noted, overall experiences were overwhelmingly negative. Ableism was pervasive. In particular, internalised ableism coloured their entire lifeworlds–if they could not pass within the neuromajority, how could they have a place in medicine? Such internalised ableism is associated with low self-esteem, reduced wellbeing, and increased feelings of isolation [39]. Some participants felt broken, others felt a need to prove they were not broken. Both mindsets fostered a sense of shame, reinforced by fear of ill-treatment if they were identified as different. Participants therefore went to great lengths to mask (or camouflage) their ADHD–sometimes this was conscious, other times not. No studies have explored masking ADHD in adulthood or its impacts. In autistic adults, however, masking has been associated with poor mental health, burnout, and suicide [40, 41]. In this study, our participants did indeed describe the vast amount of energy spent on masking their ADHD. This pervasively impacted their whole lives, in and outside of their medical studies–putting strain on their relationships, their mental health, for example. This may, in part, explain the higher rates of mental ill health that neurodivergent doctors seem to experience [42].

Participants were exhausted from the constant masking. The overbearing pressure to conform–to meet the social norms amongst neurotypical peers–and to embody the cultural norms of medicine more broadly, took its toll. This pressure increased once reaching clinical placements, through fear of weaponised professionalism. The weaponisation of professionalism is not a new phenomenon. The concept of professionalism itself is historically grounded in cultural norms associated with the white, cis-gendered, heteronormative, able-bodied male experience [43]. Our participants were at a particularly high risk of weaponised professionalism as they all had multiple intersectional identities, which has been shown to increase this risk in healthcare [44]. For example, throughout the interviews, female participants highlighted how their gender seemed to negatively impact the way they were perceived by others. Participants further alluded to this weaponization of professionalism in their recollection of experiences of OSCEs–recalling negative feedback on differences in communication styles and their sensory needs. Training OSCE examiners in neurodiversity-affirmative marking, including awareness of different neurotypes and communication styles [45], could be a potential adjustment under the UK Equality Act of 2010. Such an adjustment would be supported by the GMC, who acknowledge that a disability is not a fitness to practise concern in itself [30].

Participants felt alienated from peers, educators, and the institution, following experiences of direct discrimination and microaggressions. A mismatch in communication styles with neurotypical peers and educators also seemed to contribute to feelings of isolation. This concept is best described by the double empathy problem, often seen in the context of autism, whereby communication difficulties are due to reciprocal mismatches in communicative style between neurotypes rather than a social deficit in the non-neurotypical [46]. Therefore, had participants’ communication styles been assessed in the context of communicating with others with ADHD, these may have been deemed appropriate (or even excellent) rather than deficient. This acknowledges the effect of power on such entrenched views around right and wrong ways of communicating.

Whilst participants received negative feedback for their communication styles, a body of doctors with diverse communication styles may better meet the needs of the diverse patient population–in line with GMC Welcomed and Valued [47]. Participants strived to conform to neurotypical communication styles–another form of masking–at the expense of their mental health to avoid being outcast.

Participants felt social media negatively impacted their self-esteem with inaccurate and romanticised depictions of ADHD, which is congruent with recent studies that show over 50% of videos about ADHD on TikTok and YouTube are misleading, especially when they are produced by non-healthcare workers [48, 49]. As per our participants’ experiences, wider research also shows that social media can negatively impact psychological wellbeing and self-esteem [50]. Interestingly, in previous research, videos rated the most useful were those that focused on lived experience [50]–something that our participants also emphasised. Thus, illustrating the need for doctors with ADHD to be openly visible as role models, showing they can be successful with ADHD, not despite it. This is in keeping with previous research, which found that autistic medical students desired openly autistic role models within the medical profession [14]. Here, if our participants’ desires for positive role modelling are met, it may well steer them onto a path of increased self-confidence and reduced internalised ableism–helping them to pursue their dreams and advocate for their needs–instead of one that leads to burnout and attrition [22]. This may help students to embrace their differences, rather than suppressing them. A support network for medical students with ADHD may also contribute to positive outcomes through reducing the reported feelings of alienation and isolation.

Compassion and self-compassion are protective factors against emotional exhaustion and burnout in medical students and doctors [51]. However, our participants experienced callousness at medical school, from others and themselves. This toxic culture and its impacts have been demonstrated in other studies, likely fostered by an emphasis on competitive achievement needed for the UK ranking system following medical school graduation [12, 52]. Perhaps a shift towards collaborative learning, which participants noted to be their favourite, could nurture a more compassionate and kind medical school culture which may ultimately improve patient care [52, 53].

Their medical school’s potentially tokenistic approach to inclusion–as reported by our participants–may not be fully in keeping with the UK Medical Schools Council’s active inclusion guidance [54]. The GMC also highlights that disabled students should be consulted to find the most appropriate adjustments for their individual circumstances [30]. Despite this, and whilst our participants reported multiple attempts to advocate for their needs, they reported that their cries for help were disbelieved. Adjustments which increase accountability and focus on organisation and time management were felt to be most beneficial. Diagnosis and holistic support for participants was inaccessible without financial wealth. The GMC, however, suggests that medical schools should fund support services and referrals if it is unfeasible through the NHS–and that such services should be independent from the medical school [55]. This could provide some further protection of confidentiality, which concerned participants.

### Limitations and strengths

As this is a qualitative study, our results are not generalizable. Instead, our study presents an in-depth exploration of the experiences of a sample of mostly cis-gender females from one medical school in England. This sample was well-suited for phenomenological research, enabling intimate exploration of participants’ experiences. The study strived for an in-depth analysis of the experiences of current medical students with ADHD. Therefore, it did not include the voices of those whose ADHD may be unrecognized, nor did it include those who may have left medical school.

Others involved in participants’ subjective reports of their experiences may have different recollections of events. However, the purpose of the study is to understand the perspectives of our participants. There may also be an element of volunteer bias, whereby participants chose to volunteer because they have powerful stories to tell. However, this does not mitigate or lessen their own experiences.

This is the first study of the experiences of medical students with ADHD. Meg’s insider status was key to building rapport with participants and to engaging with and understanding their stories during the analysis. It is also worth acknowledging here that, given our insider status, there is a possibility our own subjective experiences and perceptions could have influenced which examples of participants’ reported experiences were focused on. Whilst a positivist or critical realist perspective may consider this a limitation, this may also be considered a strength through a social constructionist approach to interpretive phenomenological research [35, 50].

## Conclusion

Participants reported strengths associated with ADHD such as empathy and working well under pressure, which are highly desirable aptitudes for doctors. However, participants also reported experiences of bullying and isolation at medical school, perpetrated by doctors and peers, as well as feelings of alienation and inferiority when unable to conform on placement or in exams. From this, participants adopted survival strategies, such as masking, to avoid being ostracised. All recognised their ADHD status when their mental health deteriorated during their medical studies. Of those who disclosed their diagnosis, none were offered personalised support. Participants feared disclosure, largely due to weaponised professionalism and the effects of toxic competitiveness in medicine. They yearned for a sense of belonging. This study suggests a key starting point for medical schools is to listen to their medical students, and take action to support each individual student. To have a wider impact, medical schools should evaluate how they deliver OSCEs and consider additional training for examiners.

This study has highlighted areas where medical schools can be instrumental in cultivating an environment where medical students with ADHD can thrive, not just survive. Further research is now needed to identify which adjustments are effective and to explore the experiences of non-ADHD medical students and staff in relation to medical students with ADHD.

## Supporting information

S1 Appendix Interview topic guide. (DOCX)
