# Supplementary material for: The experiences of medical students with ADHD: A phenomenological study
Source: PLoS One. 2023 Aug 22;18(8):e0290513. doi: 10.1371/journal.pone.0290513 (PMC10443849; doi:10.1371/journal.pone.0290513)
Supplement: S1 Appendix — (DOCX) [file pone.0290513.s001.docx]

**Interview topic guide**

- **Basic demographic information – age, gender identity, current year of study, any previous re-takes at medical school (specific assignments, modules, or years).**
- **Experiences in relation to self-study**

We are interested in exploring how attention deficit hyperactivity disorder (ADHD) may have impacted self-study. We are interested in any strengths/positives, challenges, and coping strategies.

- **Experiences in relation to university teaching**

We are interested in the differences between various teaching settings – online, lecture-based, lab-based, or small group work, for example.

- **Experiences in relation to clinical learning**

We are interested in exploring how ADHD may have impacted on learning in settings. We are interested in any strengths/positives, challenges, coping strategies and/or environmental impacts.

- **Emotional and social experiences**

We are interested in how ADHD may impact social and emotional experiences at medical school. For example, we are interested in interactions between medical students with ADHD, their peers, medical school staff, and clinical staff. We are also interested in exploring experiences in relation to role models or peers with ADHD in medicine.

- **Experiences in relation to disclosure**

We are interested in whether they choose to disclose their ADHD and, if so, to whom. For example, whether they have chosen to disclose formally to the university or to staff supporting them on clinical placements.

- **Experiences in relation to teaching/learning about ADHD whilst at medical school**

We are interested in what participants have been taught regarding ADHD at medical school, and how this may have impacted them. For example, we are interested in knowing if they have learned about the strengths associated with ADHD, or if they experienced purely deficit-focused teaching.

- **Experiences in relation to medication**

We are also interested in experiences with ADHD medication. Medication is shown to reduce inattention and impulsivity but may also reduce creativity and flexibility in problem solving, which are both associated with ADHD.
